# UL34 Deletion Restricts Human Cytomegalovirus Capsid Formation and Maturation

**DOI:** 10.3390/ijms23105773

**Published:** 2022-05-21

**Authors:** Declan L. Turner, Rachel M. Templin, Adele A. Barugahare, Brendan E. Russ, Stephen J. Turner, Georg Ramm, Rommel A. Mathias

**Affiliations:** 1 Infection and Immunity Program, Department of Microbiology, Monash Biomedicine Discovery Institute, Monash University, Clayton, VIC 3800, Australia; declan.turner@monash.edu (D.L.T.); adele.barugahare@monash.edu (A.A.B.); brendan.russ@monash.edu (B.E.R.); stephen.j.turner@monash.edu (S.J.T.); 2 Ramaciotti Centre for Cryo Electron Microscopy, Monash University, Clayton, VIC 3800, Australia; rachel.templin@monash.edu (R.M.T.); georg.ramm@monash.edu (G.R.); 3 Monash Bioinformatics Platform, Monash University, Clayton, VIC 3800, Australia; 4 Department of Biochemistry and Molecular Biology, Monash University, Clayton, VIC 3800, Australia

**Keywords:** HCMV, herpesvirus, UL34, replication compartment, capsid maturation, genome packaging

## Abstract

Over 50% of the world’s population is infected with Human Cytomegalovirus (HCMV). HCMV is responsible for serious complications in the immuno-compromised and is a leading cause of congenital birth defects. The molecular function of many HCMV proteins remains unknown, and a deeper understanding of the viral effectors that modulate virion maturation is required. In this study, we observed that UL34 is a viral protein expressed with leaky late kinetics that localises to the nucleus during infection. Deletion of UL34 from the HCMV genome (ΔUL34) did not abolish the spread of HCMV. Instead, over >100-fold fewer infectious virions were produced, so we report that UL34 is an augmenting gene. We found that ΔUL34 is dispensable for viral DNA replication, and its absence did not alter the expression of IE1, MCP, gB, UL26, UL83, or UL99 proteins. In addition, ΔUL34 infections were able to progress through the replication cycle to form a viral assembly compartment; however, virion maturation in the cytoplasm was abrogated. Further examination of the nucleus in ΔUL34 infections revealed replication compartments with aberrant morphology, containing significantly less assembled capsids, with almost none undergoing subsequent maturation. Therefore, this work lays the foundation for UL34 to be further investigated in the context of nuclear organization and capsid maturation during HCMV infection.

## 1. Introduction

Human Cytomegalovirus (HCMV) is a widespread human pathogen that infects most of the global population [1]. Primary infection of healthy individuals is usually mild or asymptomatic and leads to lifelong latent infection with periodic reactivation and shedding for continued transmission [2]. HCMV reactivation can lead to serious complications for solid organ and haematopoietic transplant recipients [3] as well as the immunocompromised that includes people with human immunodeficiency virus (HIV) infections or those undergoing chemotherapy [4]. HCMV is also a leading cause of congenital birth defects. Intrauterine transmission to the fetus during primary infection can cause sensorineural hearing, vision, and cognitive impairments, as well as stillbirth in severe instances [5,6]. With no vaccine available and drug-resistant mutants to frontline treatment emerging [7], understanding the foundations of viral replication and pathogenesis is central to developing novel therapeutic strategies.

HCMV is a betaherpesvirus with a 236 kb linear DNA genome encoding >170 open reading frames [8]. The virion binds distinct cell surface receptors through the glycoprotein trimer [9,10] or pentamer [11], depending on the host cell type, with membrane fusion mediated by glycoprotein B (gB) [12]. In the cytoplasm, tegument proteins are thought to dissociate from the virion and begin reprogramming the cell, primarily by inhibiting the host’s innate immune defences [13]. The nucleocapsid traffics along the host cytoskeleton [14] to the nuclear pore complex, where the genome is injected into the nucleus [15]. Inside, the linear genome circularises, and viral gene expression commences. HCMV has a tightly controlled transcriptional program with three major kinetic classes of viral gene expression transcribed by host RNA polymerase. These are the immediate early (IE), delayed early (DE), and late (L) gene products. The principal IE gene is IE1 which is under the control of the major immediate early promoter (MIEP). IE1 disrupts the interferon response, remodels chromatin, inhibits apoptosis, and is the primary transcription factor for the expression of DE genes [16]. DE genes are primarily involved in viral DNA replication, cell cycle progression, and metabolism [17]. The HCMV genome is presumed to be replicated by a rolling circle mechanism akin to the herpes simplex virus [18]. Viral DNA replication is a prerequisite for the expression of L genes, which mostly include structural capsid, tegument and membrane glycoproteins, capsid maturation effectors, and egress modulators [19].

The viral replication compartment (RC) is an intranuclear inclusion where viral DNA synthesis, gene expression, and capsid maturation occurs. RCs emerge from approximately 24 HPI in cell culture, expand as the replication cycle progresses, and occupy most of the nucleus by 96 HPI. The canonical RC marker is the viral polymerase processivity factor UL44 [20]. Recent work has described a liquid-liquid phase transition occurring in HCMV infected cell RCs, which is induced by the UL112/113 gene products and likely describes the basis for the accumulation of viral products for efficient DNA replication and capsid maturation [21]. During herpesvirus infection, capsid assembly, maturation, and genome packaging are essential and tightly regulated processes. The process of pro-capsids forming around the scaffolding protein encoded by UL80 in the nucleus before autocatalytic degradation allows space for the viral genome to be packaged [22]. Unit length genomes are packaged and cleaved by the tripartite terminase complex composed of UL51, UL56, and UL89 [23,24,25] and the DNA packaging protein UL52 [26]. At steps along this pathway, capsids can be sealed with the scaffold intact to form B capsids, or after scaffold digestion to form empty A capsids. The mature, genome-containing capsids are termed C capsids and are destined become infectious virions (reviewed [27]). Nuclear egress of the mature genome-containing capsids relies on both host and viral processes. The viral nuclear egress complex (NEC) consisting of UL50 and UL53 is essential, as is the breakdown of the nuclear lamina by phosphorylation by UL97 kinase and likely host CDK1 to allow capsids access to the nuclear membrane [28]. Capsids traverse both nuclear membranes in the process of envelopment and de-envelopment and enter the cytoplasm for further maturation.

A hallmark of HCMV infection is the cytoplasmic viral factory termed the viral assembly compartment (vAC). It is characterised by a host-derived golgi ring that clusters host endosomes and viral UL99 around a microtubule organising centre (MTOC) [29]. Nucleocapsids exit the nucleus and accumulate tegument layers while they traffic towards the centre of the vAC. Virion envelopment occurs by inward budding on the host-derived multivesicular body (MVB) limiting membranes. The viral proteins UL71 [30,31,32], UL94 [33], and UL99 [34] have all been shown to facilitate virion envelopment. After envelopment, MVBs containing enveloped virions and vesicles traffic to the cell membrane, fuse, and release infectious virions into the extracellular space. The viral protein UL103 is essential for egress post envelopment; however, the exact mechanism is not established [35]. Virion envelopment and egress are dependent on host pathways and architecture, a process that shares many parallels to exosome biogenesis [36].

Due to an interest in identifying novel viral proteins involved in virion assembly and maturation, we concatenated HCMV gene information to focus on a subset of gene products. This approach uncovered a cluster of viral modulators containing late viral transcriptional regulators, DNA packaging proteins, and UL34.

## 2. Results

### 2.1. Characterisation of HCMV Genes by RNA Sequencing and Bioinformatic Analysis

**Figure 1:**
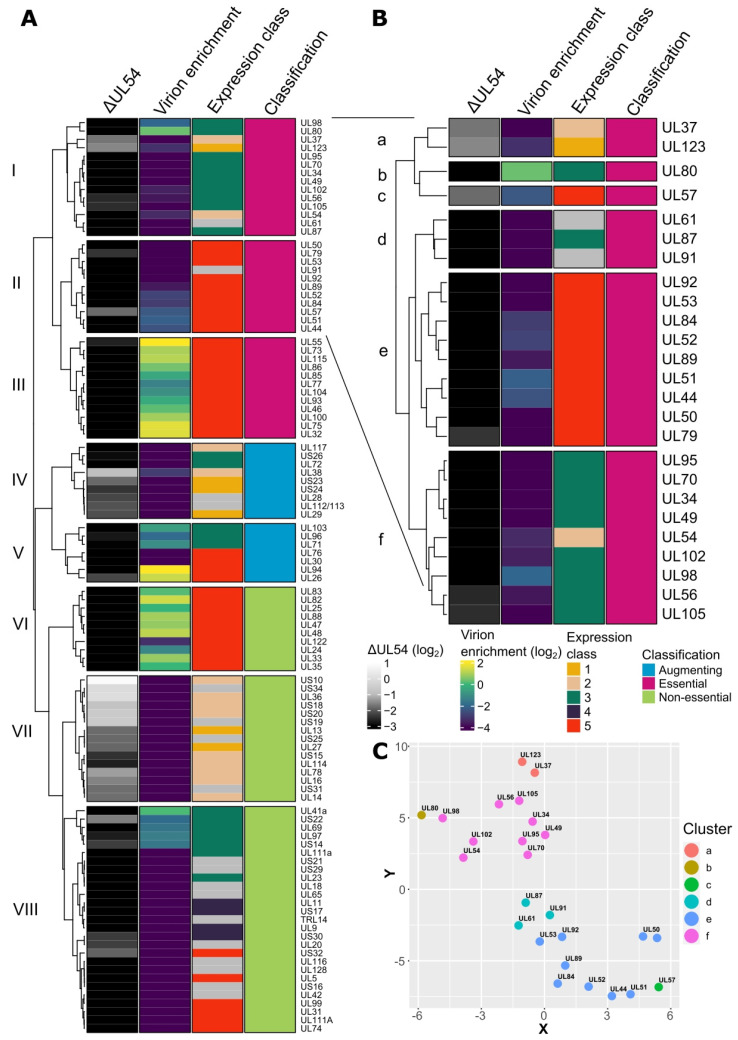
RNA sequencing and bioinformatics analysis of HCMV genes. (**A**) Heatmap depicting HCMV gene features based on complete linkage clustering and Gower distance. Columns represent the relative log_2_ fold-change in transcript abundance in ΔUL54 mutant infections compared to WT at 72 HPI, relative log_2_ virion enrichment compared to a WT infected cellular lysate at 5 DPI [36] (positive values reflect virion enrichment), kinetic expression classes 1 to 5 based on Weekes et al. [19] and essential/augmenting/non-essential gene classification based on Yu et al. [37]. (**B**) Heatmap depicting HCMV genes expanded from clusters I and II in (**A**), and sub-clustered based on complete linkage clustering and Gower distance. (**C**) t-stochastic neighbour embedding plot of genes from (**B**).

We set out to identify important HCMV genes modulating the late stages of the viral replication cycle. Specifically, viral genes robustly expressed post-viral DNA replication and are not enriched in the mature virion. To do this, we conducted RNA sequencing for cells infected with the WT virus or a mutant virus lacking the viral DNA polymerase (ΔUL54) (Figure S1A, Supplementary File S1) and leveraged our previous proteomics-based analysis of purified virion preparations and infected cell lysates [36]. Kinetic expression classes one to five were based on Weekes et al. [19], where classes one and two are expressed early and independently of viral DNA replication. Additionally, classes three to five follow viral genome replication with some differences and nuances, and finally, essential/augmenting/non-essential classification was based on Yu et al. [37]. These datasets were combined to cluster HCMV genes employing Gower distance [38,39], with silhouette width to optimise the number of clusters [40] (Figure S1B), and a heatmap plotted to summarise all data sets within eight main clusters (Figure 1A). The clustering was further confirmed by t-stochastic neighbour embedding (t-SNE) analysis [41] using Gower distance (Figure S1C).

Clusters VII and VIII contained non-essential genes products separated by expression kinetics (Figure 1A and Figure S1C). Cluster VI was enriched with non-essential tegument proteins with class five expression (late) and high virion enrichment. These included UL25 [42], UL82 [43], UL83 [13], and UL88 [44] (Figure 1A). Clusters IV and V grouped augmenting gene products with a diverse set of known functions (Figure 1A). For example, UL71, UL94, and UL103 are involved in the late cytoplasmic stages of envelopment and egress [30,31,32,33,35], UL26 is delivered as virion cargo to augment the host response [45,46], and UL112/113 is expressed early and facilitates RC formation [21]. Cluster III contained gene products with essential classification and class five expression kinetics (Figure 1A and Figure S1C). Typically, during WT infection, these genes are translated to become virion constituents and include structural capsid proteins UL46/TRX1, UL77/CVC2, UL85/TRX2, UL86/MCP, UL93/CVC1, and UL104, along with envelope glycoproteins UL55/gB, UL73/gN, UL75/gH, UL100/gM, and UL115/gL. Cluster I and II genes also had essential classification, mostly class 3 or 5 kinetics, but were not enriched in virion cargo following their translation in WT infections (Figure 1A and Figure S1C).

Gene products in clusters I and II were further sub-clustered into six clusters (Figure S1D and Figure 1B) and confirmed with t-SNE analysis (Figure 1C). Interestingly, sub-clusters d, e, and f were only separated based on class three or five expression kinetics (with the exception of UL54), both of which are considered late and have substantial overlap (Figure 1B,C) [19]. These sub-clusters contained 6/6 viral pre-initiation complex (vPIC) subunits (UL49, UL79, UL87, UL91, UL92, and UL95) [47,48,49], both NEC subunits (UL50 and UL53) [50], the viral DNA polymerase UL54/POL, all sub-units of the tripartite helicase-primase complex (UL70, UL102, and UL105) [51], all subunits of the tripartite terminase complex (UL56/TRM1, UL51/TRM2, and UL89/TRM3) [23,24,25] and the cleavage packaging protein UL52 [26] (Figure 1B,C). Additionally, the uncharacterised protein UL61 and UL34, which has ambiguous functional characterisation, were present in these clusters (Figure 1B,C). Given that UL61 is in proximity to the origin of lytic replication in the HCMV genome and was not detected by mass spectrometry [19], further investigation was omitted. Additionally, UL34 was robustly detected by mass spectrometry [19] and was previously shown to be essential for viral growth in two independent studies [37,52]. Therefore, UL34 was selected for further functional characterisation.

### 2.2. UL34 Is an Augmenting HCMV Gene Not Required for Viral Genome Replication or vAC Formation

The ΔUL34 mutant AD169 BAC was constructed using site-directed mutagenesis by Yu et al. [37]. The *UL34* gene has ATG codons at nucleotides 1–3 and at 64–66, which may act as an alternative start site for translation. A transposon encoding selectable markers were inserted into the 5′ region of *UL34*, deleting nucleotides 1 to 66 ensuring no functional truncated protein could be translated. We verified the transposon insertion by blue-white screening, PCR, and Sanger sequencing (Figure S2A–C) and generated a ΔUL34 viral stock by electroporating the ΔUL34 BAC into complementing fibroblasts stably expressing UL34 with an n-terminal HA tag and linker. In addition, we recombineered an AD169 BAC to tag UL34 with an n-terminal HA-tag and linker.

**Figure 2:**
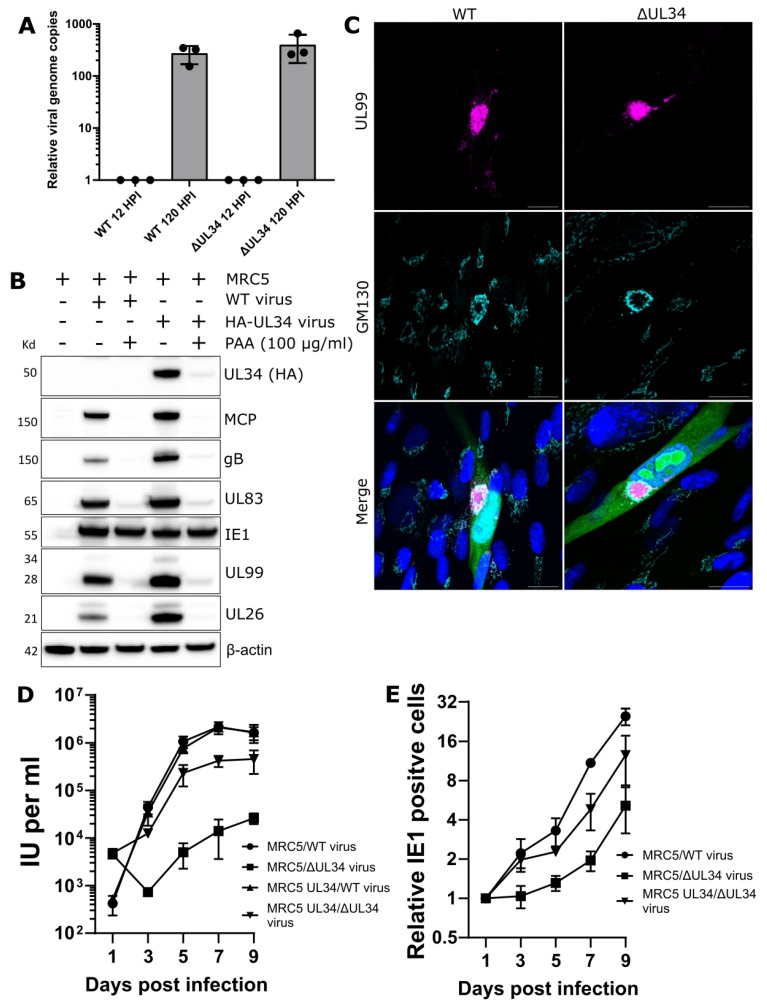
Characterisation of UL34 as an augmenting viral protein with leaky late expression kinetics. (**A**) Intracellular HCMV genome copies at 120 HPI, relative to genome copies at 12 HPI. MOI = 3, n = 3, bars = SD. (**B**) Western blot analysis of UL34 expression in MRC5 cells treated with 100 μg/mL phosphonoacetic acid (PAA) and subsequently infected with either AD169-GFP WT or AD169 HA-UL34 HCMV (5 DPI, MOI = 3). Membranes were probed with primary antibodies against HCMV viral proteins, HA, or β-actin loading control. (**C**) Immuno-fluorescence analysis of host GM130 and viral UL99 in WT MRC5 cells infected with WT or ΔUL34 AD169-GFP virus. 4 DPI, MOI = 0.1, scale bars = 20 μm. (**D**) Growth kinetics of ΔUL34 AD169-GFP virus, as measured by IE1 fluorescent focus assay in cell culture supernatants from WT and UL34-complementing MRC5 cells. MOI = 3, n = 3, bars = SD. (**E**) Spread of ΔUL34 AD169-GFP virus in WT and UL34-complementing MRC5 cells, as quantified by fixing, staining, and counting IE1 positive cells at indicated time points. MOI = 0.01, n = 3, bars = SD.

Firstly, we assayed viral genome replication by qPCR at 12 and 120 HPI. Comparing time-points, cells infected with either WT or ΔUL34 virus had 273 or 400-fold increases in viral DNA copies, respectively (Figure 2A). This confirmed that UL34 is dispensable for viral DNA replication. WT cells were treated with the potent viral polymerase inhibitor phosphonoacetic acid (PAA) to verify that UL34 expression required viral DNA replication, and infected with endogenously tagged HA-UL34 virus. Western blotting confirmed minimal UL34 expression in the absence of viral DNA replication (Figure 2B). Finally, vAC formation was assessed in ΔUL34 infected cells at 96 HPI, revealing a prototypical vAC with golgi ring and UL99 localisation (Figure 2C). These experiments validated the progression of the replication cycle to late stages in the absence of UL34.

Next, a high multiplicity of infection (MOI) growth analysis showed a 2-log_10_ fold reduction in extracellular virus production for ΔUL34 virus, with substantial but incomplete rescue in HA-UL34 expressing cells (Figure 2D). Similarly, a low MOI spread assay confirmed a 7-fold reduction with ΔUL34 virus at seven and nine days post-infection (DPI) (Figure 2E). Taken together, UL34 is an augmenting gene not required for DNA replication or vAC formation, but its deletion from the genome significantly reduces (100-fold) the production of extracellular virions.

### 2.3. UL34 Is Expressed with Leaky Late Kinetics and Localises to the Nucleus

**Figure 3:**
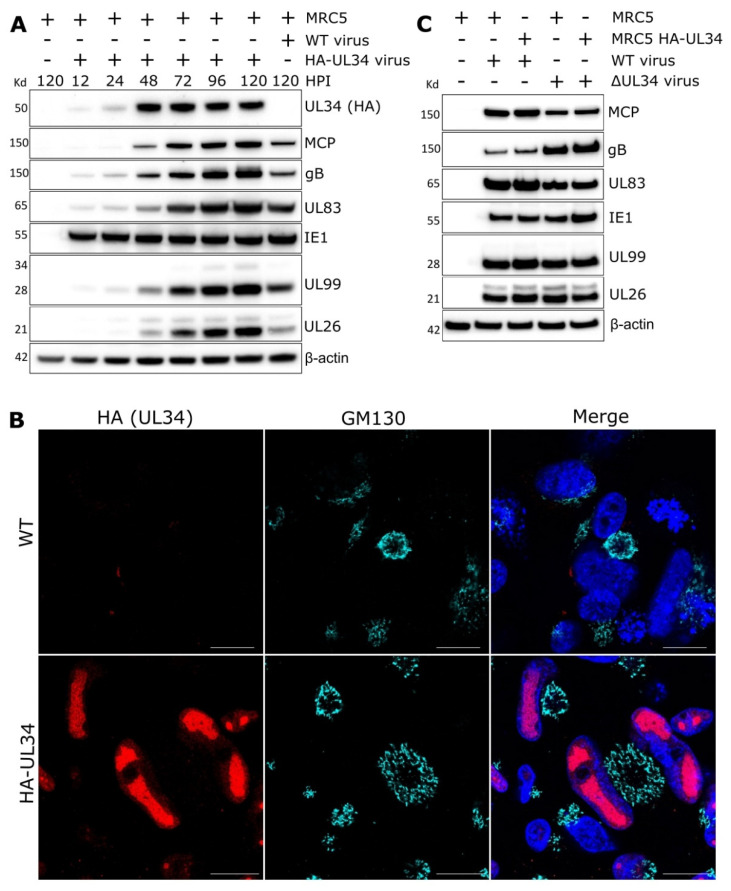
Characterisation of UL34 as a nuclear viral protein dispensable for viral gene expression. (**A**) Western blot analysis of UL34 expression kinetics. MRC5 cells were infected with HCMV AD169 HA-UL34 (endogenous n-terminal HA tag and linker) virus and lysed at various times post-infection. Mock and WT AD169-GFP WT control conditions were lysed at 5 DPI (MOI = 3). Membranes were probed with primary antibodies against HCMV viral proteins, HA, or β-actin loading control. (**B**) Immuno-fluorescence analysis of MRC5 cells infected with AD169-GFP or AD169 HA-UL34 HCMV and stained with HA (UL34) and GM130 antibodies. 4 DPI, MOI = 0.1, scale bars = 20 μm. (**C**) Western blot analysis of lysates from MRC5 and UL34 complementing cells infected with either WT or ΔUL34 HCMV AD169-GFP virus. 5 DPI, MOI = 3. Membranes were probed with primary antibodies against HCMV viral proteins or β-actin loading control.

The expression kinetics of UL34 were examined over a period of 5 DPI. A weak UL34 signal was detected at 12–24 HPI, with robust expression present at 48–120 HPI (Figure 3A). These kinetics are consistent with expression following viral genome replication, but earlier than true late proteins, such as major capsid protein (MCP) and envelope glycoprotein B (gB).

The localisation assessment of the endogenously tagged HA-UL34 virus at 96 HPI revealed intranuclear localisation (Figure 3B). Staining appeared absent from the nuclear periphery and consistent with viral replication compartments where viral genomes are replicated and capsids assembled [20,53,54]. Further time-course analysis between 24–96 HPI confirmed that from 48 HPI, when UL34 is robustly expressed (Figure 3A), UL34 localises to intranuclear puncta that enlarge to occupy most of the nuclear area by 96 HPI (Figure S3A).

Given the nuclear localisation and expression kinetics of UL34, we investigated whether ΔUL34 infection impacted the expression of other viral proteins. Compared to WT, western blotting analysis of lysates from cells infected with ΔUL34 virus did not reveal differential expression of proteins across expression and functional classes, including Immediate early protein 1 (IE1), MCP, gB, or tegument proteins UL26, UL83, and UL99 (Figure 3C). UL34 has been previously reported to function as a transcriptional repressor and regulate other viral transcripts [55]. We observed elevated expression of non-essential US3 and US9 [52] transcripts in ΔUL34 infections compared to WT (Figure S3B). However, expanded analysis of transcripts encoding essential virion maturation regulators and structural virion components revealed no significant difference (Figure S3C). We reasoned that a sole transcriptional repression function could not entirely explain the 100-fold reduction in virus we measured in ΔUL34 infections and that an alternative mechanism could be possible.

### 2.4. UL34 Interacts with Host Nuclear Regulators but Does Not Modulate Lamina Integrity

**Figure 4:**
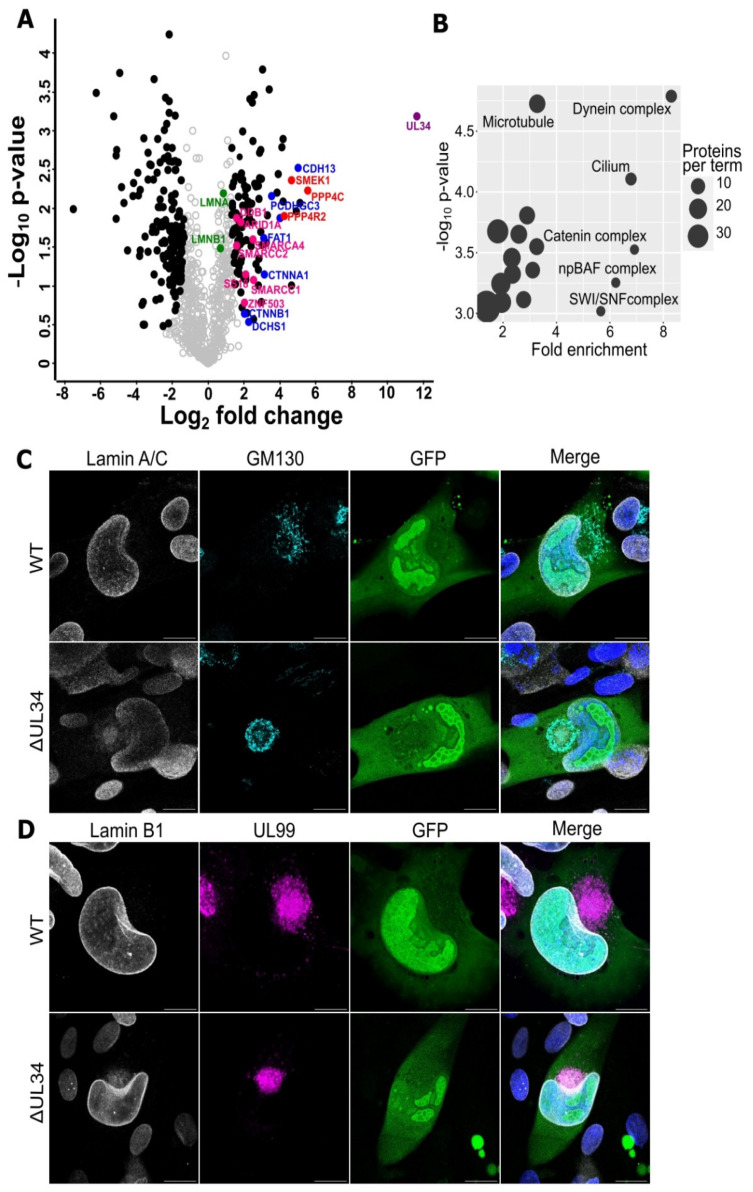
Analysis of UL34 protein interactions. Raw spectral files from UL34 immunoprecipitation (IP) experiments generated by Nobre et al. [56] were downloaded and researched using MaxQuant. (**A**) Volcano plot showing relative enrichment of proteins in the UL34 IP (positive fold change), compared to control. Purple: UL34 bait, Blue: cadherins, Green: nuclear lamins, Pink: chromatin modifiers, Red: PP4 phosphate complex sub-units, Black circles: significant differential expression. n = 2, S_0_ = 2, FDR < 0.05. (**B**) Bubble plot depicting significantly enriched Gene Ontology terms from the list of UL34 interactors, compared to background (*p* < 10^−3^). The x-axis represents the fold enrichment of the term in the target list compared to the expected number based on the background. Bubble size represents the number of IDs associated with each term. (**C**) Immuno-fluorescence analysis of GM130 and lamin A/C in WT MRC5 cells infected with WT or ΔUL34 AD169-GFP virus. 4 DPI, MOI = 0.1, scale bars = 20 μm. (**D**) Immuno-fluorescence analysis of GM130 and lamin B1 in WT MRC5 cells infected with WT or ΔUL34 AD169-GFP virus. 4 DPI, MOI = 0.1, scale bars = 20 μm.

A previous HCMV interactome by Nobre et al. [56] revealed that UL34 interacted with the PP4 serine/threonine phosphatase complex subunits and the non-canonical proto-cadherins. To explore this data further, we downloaded and re-searched the raw spectral files using our analysis pipeline. Initial principal component analysis separated the control immunoprecipitation (IP) samples from the UL34 IPs (Figure S4A), and relative quantitative comparison identified significant protein interactions (Figure 4A). Subsequent gene ontology (GO) enrichment analysis revealed “microtubule,” “SWI/SNF complex,” “cilium,” “catenin complex,” and “dynein complex” to be the most enriched terms (Figure 4B) associated with the interacting proteins. Of interest, and further elaborated upon in the discussion, were the SWI/SNF complex subunits and their regulators (Figure 4A, pink dots), as well as the catenin complex and associated cadherins (Figure 4A, blue dots). The high enrichment of the PP4 phosphatase complex (Figure 4A, red dots), as well as the moderate enrichment of nuclear lamins A/C and B (although failing to achieve the threshold for statistical significance (Figure 4A, green dots)), was also interesting. PP4 negatively regulates CDK1 activity [57], which phosphorylates nuclear lamins at the same residues as the viral kinase UL97 to promote lamina disassembly [58], enabling the egress of the genome-containing capsids into the cytoplasm [59].

Lamin A/C and B1 morphology were imaged in cells infected with ΔUL34 or WT virus at 4 DPI to investigate the involvement of UL34 in nuclear lamina breakdown. However, no discernible difference was observed (Figure 4C,D). Similarly, although HCMV infection increased the overall levels of phosphorylated lamin A/C, no difference was observed when WT and ΔUL34 infections were compared (Figure S4B). Finally, staining infected cells for UL34 and lamin B1 revealed no co-localization (Figure S4C). Instead, UL34 appeared to localise within replication compartments based on a concentrated GFP signal (Figure S4C), and the replication compartment morphology in ΔUL34 infected cells had a vacuolated, “honeycomb” appearance compared to cells infected with WT virus, which was mostly uniform (Figure 4C,D).

### 2.5. Cells Infected with HCMV Lacking UL34 Display Reduced Capsids That Fail to Mature

**Figure 5:**
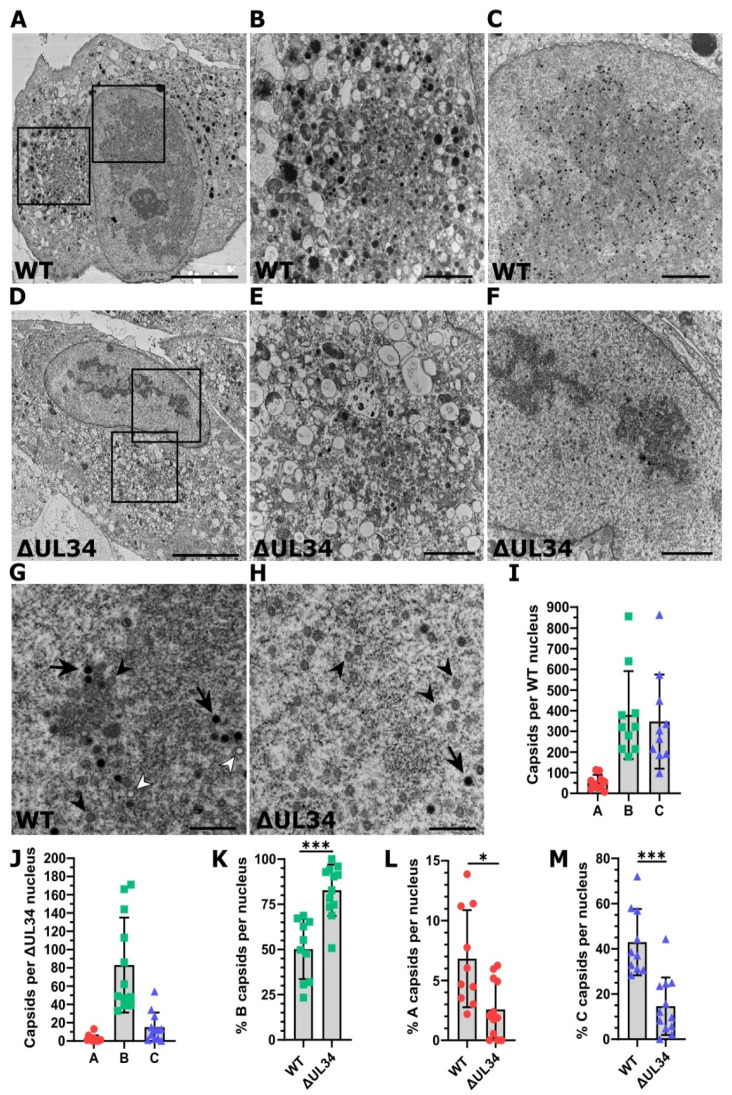
TEM analysis of cells infected with WT HCMV or ΔUL34 virus. (**A**) Representative electron micrograph of an MRC5 cell infected with WT AD169-GFP virus. 5 DPI, MOI = 1, scale bar = 10 μm. (**B**,**C**) Inset areas from (**A**) show the cytoplasmic vAC and nucleus, respectively. Scale bars = 2 μm. (**D**) Representative electron micrograph of an MRC5 cell infected with ΔUL34 AD169-GFP virus. 5 DPI, MOI = 1, scale bar = 10 μm. (**E**,**F**) Inset areas from (**D**) show the cytoplasmic vAC and nucleus, respectively. Scale bars = 2 μm. (**G**,**H**) Representative electron micrographs depicting the distinct capsid types in the nuclei of MRC5 cells infected with WT or ΔUL34 AD169-GFP virus, respectively. White arrowhead: A capsids, Black arrowhead: B capsids, Black arrow: C capsids, 5 DPI, MOI = 1, scale bars = 500 nm. (**I**) Bar chart depicting the total number of each capsid type (A, B or C) in WT infected nuclei cross-sections. Bars = SD, n = 10. (**J**) Bar chart depicting the total number of each capsid type in ΔUL34 infected nuclei cross-sections. Bars = SD, n = 12. (**K**–**M**) Percentage of B, A, and C capsids in WT or ΔUL34 infected nuclei cross-sections shown in (**I**,**J**). WT n = 10, ΔUL34 n = 12, bars = SD, * *p* < 0.05, *** *p* < 0.001, two-tailed *t*-test with Welch’s correction.

To investigate nuclear events with greater resolution, we performed transmission electron microscopy on cells infected with WT or ΔUL34 virus 5 DPI (Figure 5). In WT infections, maturing virions were present in the cytoplasmic vAC, and an abundance of capsids was visible within the kidney bean-shaped nucleus (Figure 5A–C). By contrast, the cytoplasm of ΔUL34 infected cells was almost completely devoid of viral activity in general, and the nuclear replication compartment appeared more electron-dense with discontinuous areas (Figure 5D–F). In addition, while some maturing capsids were observed, the overall number was significantly reduced.

WT and ΔUL34 infected cell nuclei were imaged at high magnification, and sequential images merged to produce high-resolution images to assess each capsid type (Figure 5G,H). B and C capsids in WT infected cells were observed in approximately equal proportion, with fewer A capsids (Figure 5G,I). By contrast, ΔUL34 infected cells contained fewer capsids overall and were dominated by B capsids, with significantly fewer C capsids and almost no A capsids (Figure 5H,J). We also evaluated the proportions of each capsid type. Compared to WT, ΔUL34 infections had a significantly higher proportion of B capsids (83% to 50%) (Figure 5K), a significantly lower proportion of A capsids (2.6% to 6.8%) (Figure 5L), and C capsids (15% to 43%) (Figure 5M). We conclude that infections with ΔUL34 virus produce significantly fewer total capsids that display a capsid maturation defect within nuclear viral replication compartments where UL34 localises.

## 3. Discussion

Virion assembly and egress is a complex biological process driven by distinct kinetic viral gene products and requires the cohesion of multiple host-remodelled organelles. This study harnessed new and existing datasets to cluster and assigned HCMV gene products. This analysis provided insights across the HCMV genome. We used this resource to identify late-stage genes that may function after viral DNA replication but were not structural virion components. Using this framework, we identified the enigmatic viral proteins UL61 and UL34 and further characterised the latter.

Previous UL34 classification based on viral spread following electroporation of the BAC genome reported UL34 to be essential for growth [37,52]. However, we found UL34 to be augmenting, as ΔUL34 infections were able to spread but produced 100-fold less extracellular virus in cell culture (Figure 2D,E). Our results were obtained using a ΔUL34 virus stock derived from a UL34 complementing cell line and are consistent with similar results recently reported for M34 in MCMV [60]. Our results were obtained using the serially passaged AD169 strain in fibroblasts and await further validation in other HCMV strains and cell types. We observed robust expression and intranuclear localisation of UL34 from 48 HPI (Figure 3A,B and Figure S3A), consistent with viral RCs [60,61,62]. UL34 has been previously suggested to impact viral DNA replication efficiency [62]. In that study, mutation of UL34-binding sequences near the origin of lytic replication resulted in viral replication defects. However, viral DNA replication was not directly quantified in the absence of UL34. In the present study, high MOI ΔUL34 viral infections did not reduce viral DNA levels at the late time point assayed (Figure 2A). In addition, using a transient transfection-based experimental design, UL34 has been reported to repress gene expression from early promoters before 24 HPI [55,63,64], and we confirmed increased levels of US3 and US9 in the absence of UL34 (Figure S3B). UL34 has also been suggested to increase gene expression globally across IE, E, and L kinetic expression classes using an electroporation-based approach [61]. However, we observed negligible alterations to levels of several essential viral proteins or transcripts from most expression and functional classes in ΔUL34 infections, including the tripartite terminase, capsid components, tegument, and gB (Figure 3C and Figure S3C). A limitation of our study is that the genome replication assay (Figure 2A), and transcript/protein expression measurements (Figure 3C and Figure S3B,C), were conducted at a single late time point. It is possible that differences in these measurements may exist at earlier time points. However, given the large (100-fold) reduction in virus titre (Figure 2D,E), the minimal expression of UL34 before 48 HPI (Figure 2B and Figure 3A) [19] and clear RC morphology and capsid maturation phenotype observed (Figure 4C,D and Figure 5A–M), the most parsimonious interpretation is that UL34 contributes to the replication cycle at late stages of infection. Therefore, our working hypothesis is that viral gene regulation is not the primary function responsible for the augmenting phenotype we observed in ΔUL34 infections.

The HCMV RC is an essential structure for viral DNA replication, capsid assembly, and genome cleavage-packaging [20,53,54]. We observed WT RCs to be large and mostly uniform (Figure 5A,C). In contrast, ΔUL34 infections displayed aberrant RC morphology and instead presented as smaller, more electron-dense regions (Figure 5D,F) that excluded GFP localisation (Figure 4C,D). In WT infected cells, all capsid types, particularly C capsids, localised around the periphery of RCs (Figure 5A,C,G), consistent with reports that DNA is selectively cleaved and packaged at the RC periphery [65]. This may represent the boundary between tightly wound heterochromatin and loose euchromatin, which is a dynamic zone of chromatin remodeling, gene regulation, and transcription [66]. In ΔUL34 infections, capsids were dispersed throughout the nucleus, including in regions close to the nuclear envelope (Figure 5F,H). Herpesviral genomes are known to be chromatinised during both lytic and latent infection (reviewed [67]), but histones are absent from capsid-packaged genomes [68,69,70]. To date, no specialist herpesviral protein has been reported to regulate chromatin status during infection, although IE1 has been shown to modify nucleosome occupation on viral DNA [69,71]. Interestingly, our analysis of UL34 interaction partners identified the enrichment of the SWI/SNF complex (Figure 4A,B), a canonical regulator of nucleosome sliding and disassembly [72]. Additionally, members of the catenin complex known to translocate to the nucleus and remodel chromatin through the SWI/SNF complex [73,74] were also present in UL34 interactions (Figure 4A,B), as was the PP4 phosphatase complex that is associated with nucleosome disassembly [75,76]. Based on the UL34 interaction network together with the RC phenotype observed in ΔUL34 infection (Figure 4C,D and Figure 5A–H), future studies could investigate the involvement of UL34 in nucleosome disassembly, regulating correct RC architecture and capsid localization, and efficient packaging of viral genomes.

Cells infected with the ΔUL34 virus were able to express similar levels of MCP as WT (Figure 3C and Figure S3C). However, ultrastructural analysis revealed fewer total capsids per nucleus in ΔUL34 infections compared to WT (Figure 5I,J). In addition, a reduced percentage of A and C capsids compared to B capsids was observed (Figure 5K–M). This phenotype is consistent with infections lacking the terminase [23,24,25] or UL52 [26] proteins or blocked with the terminase inhibitor BDCRB [77]. Similarly, deletion of the capsid vertex components 1 and 2 (UL77 and UL93) also results in B capsid accumulation [78]. Given that these transcripts were present at similar levels in WT and ΔUL34 infections (Figure S3C), the capsid maturation defects observed are not due to the down-regulation of essential capsid maturation gene expression (Figure 5A–M). As the precise molecular details describing capsid assembly and maturation are emerging [79], with the phenotype observed in ΔUL34 infection, UL34 may be involved in these processes.

In summary, we integrated new and existing datasets to identify UL34 as a viral protein that functions at late stages of the replication cycle. We discovered that UL34 deletion reduces viral titres by 100-fold and is dispensable for viral DNA replication. The ΔUL34 infections display altered RC morphology and reduced capsid formation. This work lays the foundation for future work to investigate the precise mechanistic function of UL34 during HCMV capsid assembly and maturation.

## 4. Methods

### 4.1. Cells and Viruses

MRC5 (ATCC CCL-171) primary fetal lung fibroblasts and HEK293T (ATCC CRL-3216) embryonic kidney cells were purchased from the ATCC and cultured in DMEM (Thermo Fisher Gibco, Waltham, MA, USA) supplemented with 10% (*v*/*v*) Fetal Bovine Serum (Cell Sera, Rutherford, Australia), 10 U/mL penicillin, and 10 U/mL streptomycin (Thermo Fisher Gibco, Waltham, MA, USA). Cells were maintained in a humidified incubator at 5% CO_2_, 37 °C, and passaged 1:3 to 1:5 for MRC5 or 1:10 for HEK293T every third day.

HCMV was reconstituted by electroporating bacterial artificial chromosomes (BACs) containing the AD169-GFP genome and pp71 expression plasmid into MRC5 cells. AD169-GFP and ΔUL34 BACs were kindly provided by Prof. Thomas Shenk [37,80]. Infected cellular supernatant was collected, clarified by centrifugation at 1500× *g*, and underlaid with a sorbitol cushion (20% (*m*/*v*) D-sorbitol, 1X PBS, pH 7.4). The media was centrifuged at 50,000× *g* for 1 h at 4 °C [81]. Virus pellets were resuspended in full media, aliquoted, stored at −80 °C, and titred by IE1 fluorescent focus assay (described below). Cell monolayers were seeded 24 h prior and infected with HCMV in a low volume of media (adjusted depending on well format) at the indicated MOI, depending on the experiment, for 2 h at 37 °C with frequent agitation. After removal of the inoculum, fresh growth medium was added, and the cells were incubated for the indicated time depending on the experiment.

### 4.2. Cloning and Stable Cell Line Generation

HCMV gene inserts were amplified from isolated BAC DNA using Phusion high fidelity polymerase (Thermo Fisher Scientific, Waltham, MA, USA) with primer extensions complementary to pBMN-HA (UL34) or pBMN puro (UL54) vectors (provided by Dr. Michael Lazarou). Plasmids were constructed using the Gibson Hi-Fi cloning kit (NEB, Ipswich, MA, USA) according to the manufacturer’s instructions. Colonies were PCR screened with a pBMN forward sequencing primer and gene-specific reverse Gibson primer for correct inserts. Inserts were further validated by restriction digest with BamHI and SalI (UL54) or HindIII (UL34). All inserts were Sanger sequenced prior to use for cell line generation. Primer sequences used can be found in Supplementary File S2.

To generate stable cell lines, inserts containing plasmids were isolated (PureLink plasmid miniprep kit, Invitrogen, Waltham, MA, USA). Additionally, 4 × 106 HEK293T cells (mycoplasma negative by PCR) transfected with 2 μg VSV-G, 4 μg gag/pol (retrovirus) packaging vector, and 6 μg pBMN plasmid containing inserts using Lipofectamine 3000 (Invitrogen, Waltham, MA, USA), according to manufacturer’s instructions were isolated. Approximately 16 h post-transfection, the culture medium was changed. After 24 h, the supernatant containing retroviral particles was syringe-filtered through 0.45 μm Acrodisc filters (Pall, Port Washington, NY, USA) and added directly to the cells (two harvests, 24 h apart). Next, pBMN-UL54 cells were placed under selection using 3 μg/mL puromycin for 5 days and changed at 48 h intervals. The pBMN HA-UL34 does not contain a selectable marker; however, a transduction efficiency of >80% was achieved. Cells were passaged as per ‘cells and viruses.’

### 4.3. IE1 Fluorescent Focus and Spread Assay

IE1 fluorescent focus assay was performed as previously described [36]. Briefly, the infected cell supernatant was collected, and centrifugation was performed at 500× *g* for 5 min to remove cell debris. Low-speed centrifugation was not used for virus stock titres, as they were clarified during the isolation protocol. Next, 1:4 serial dilutions were performed using full media, starting from neat to 4^−5^ for growth curves and 4^−2^ to 4^−12^ for virus stocks. 100 μL of each dilution was added to a 96-well reporter plate of confluent uninfected MRC5 cells. Cells were fixed and stained 24 HPI with mouse anti-IE1 primary (1:100, Clone 1B12 [82]) and Hoechst nuclear stain as per “Immunofluorescence confocal microscopy.” Reporter plates were imaged automatically using a DMi8 (Leica, Wetzlar, Germany) microscope with 10 × objective. A focus map was constructed with a single point per well using the Hoechst channel in LAS X navigator (Leica, Wetzlar, Germany). A 3 × 3 tilescan was performed with 0% image overlap and a fill factor of 75% per well for both Hoechst and IE1 channels. IE1 foci were viewed in the LAS X core offline version (Leica, Wetzlar, Germany) and manually counted at appropriate dilutions for IU/mL calculations. For the spread assay, confluent MRC5 fibroblasts were infected as per “Cells and viruses” at a multiplicity of 0.01 and fixed periodically between 1 and 9 DPI. Plates were stained for IE1, imaged, and the total IE1 expressing cells were quantified as shown above.

### 4.4. BAC Recombineering

The AD169-GFP ΔUL34 BAC is maintained in *E. coli* DY380 encoding lambda red recombination genes under a temperature-inducible promoter. The ΔUL34 BAC contains the YD-Tn1721 transposon insertion in the n-terminus with the first 22 aa of the *UL34* gene deleted [37] (Figure S1A–C). As the transposon already encodes kan and *lacZ* selectable markers, only a single recombination step and the negative screen is required to introduce the n-terminal HA tag. A forward primer with 70 bp homology to the UL34 promoter region and 25 bp homology to the HA-tag of pBMN HA-UL34 and a reverse primer at position 358 of the *UL34* gene were designed. A 428 bp PCR product was generated from the pBMN HA-UL34 plasmid using Phusion high fidelity polymerase (Thermo Fisher Scientific, Waltham, MA, USA) and gel extracted (Promega, Madison, WI, USA). The PCR product has 70 bp homology upstream of the UL34 START codon and 235 bp homology downstream of the transposon.

Recombineering was performed according to the standard protocol previously described [83]. Briefly, DY380 *E. coli* containing the ΔUL34 AD169 BAC were grown overnight at 32 °C. A 300 μL O/N culture was added to 17.5 mL LB with 25 ug/mL chloramphenicol and grown at 32 °C with shaking for 2 h in duplicate. After 2 h, Lambda prophage was induced at 42 °C for 15 min in a shaking water bath. The second tube was left at 32 °C as the uninduced control. Both induced and uninduced cultures were cooled in an ice slurry for 10 min. The bacteria were pelleted at 2000× *g* for 10 min, resuspended in 30 mL ice cold MilliQ, centrifuged as before, and resuspended in 1 mL ice cold MilliQ. The resuspended cells were transferred to a 1.5 mL centrifuge tube, washed twice more as above in 1 mL ice cold MilliQ, then resuspended in a final volume of 200 μL ice cold MilliQ. Then, 1.2 ul of the HA-UL34 PCR product was added to 50 μL of induced and uninduced *E. coli*. The cells were electroporated at 1.8 kV, 25 μF, and 200 Ω with a time constant of 5.1 ms in 1 mm gap cuvettes (Bio-Rad, Hercules, CA, USA). Samples were recovered with shaking for 2 h in LB at 32 °C, then spread on X-gal plates containing chloramphenicol and incubated for 24 h at 32 °C.

Colony PCR was performed on all white colonies using the UL34 forward promoter and internal UL34 reverse primers. White colonies lacking the transposon were streaked a second time on X-gal plates, and single colonies were used to prepare glycerol stocks. PCR using Phusion polymerase was on positive clones, and the products were sent for Sanger sequencing to confirm the HA tag and UL34 ORF had the correct sequence and were in the frame. Virus stocks of HA-UL34 AD169 virus were reconstituted in WT MRC5 cells as per ‘Cells and Viruses.’

### 4.5. Densitometry-Based Protein Quantification

Cells were washed once with PBS and lysed in LDS buffer (141 mM Tris, 2% (*w*/*v*) LDS, 10% (*v*/*v*) glycerol, 0.51 mM EDTA, 0.22 mM G250, 50 mM DTT, 50 mM TCEP, 50 mM chloroacetamide, pH 8.5) on ice for 15 min, tip sonicated, and heated for 10 min at 95 °C. Then, 5 μL of samples and BenchMark protein standard (Invitrogen, Waltham, MA, USA) were subjected to PAGE (NuPAGE 4–12% Bis-Tris gels, 140 V). The gels were stained with SYPRO ruby (ThermoFisher Scientific, Waltham, MA, USA) according to manufacturer’s instructions (quick protocol). The gels were imaged at a resolution of 200 μm, with 470 nm excitation and 610 nm emission filters set using a Typhoon Trio (GE Healthcare, Chicago, IL, USA). The protein concentration in each sample was determined by relative densitometry compared to the standard using ImageQuant analysis toolbox software (GE Healthcare, Chicago, IL, USA).

### 4.6. Western Blotting

Samples were subjected to PAGE as before, and proteins wet transferred to PVDF membranes using a Mini-PROTEAN Tetra cell (BioRad, Hercules, CA, USA) at 120 V for 60 min at 4 °C in Tris-glycine buffer. Membranes blocking was performed in 5% (*w*/*v*) skim milk in 1X TBST (150 mM NaCl, 50 mM Tris pH 7.4, 0.1% (*v*/*v*) Tween 20) at 4 °C for 1 h, membranes were incubated with primary antibodies: anti-UL99 (Clone 10B4 [34]), anti-UL83 (Clone 8F5 [84]), anti-MCP (custom ordered rabbit polyclonal, Cusabio, Houston, TX, USA), anti-HCMV gB (ab6499, Abcam, Cambridge, UK), anti-IE1 (Clone 1B12 [82]), anti-UL26 [45], anti-HA (ab130275, Abcam, Cambridge, UK), mouse anti-lamin A/C (4777T, Cell Signaling Technology, Danvers, MA, USA), rabbit phospho-lamin A/C (ser-22) (13448T, Cell Signaling Technology, Danvers, MA, USA) and anti-β-actin (A2228, Sigma, Burlington, MA, USA). Primary antibodies were diluted 1:1000 in 5% skim milk and incubated overnight at 4 °C. Membranes were washed three times with TBST, and anti-rabbit (#1706515, Bio-Rad, Hercules, CA, USA) or anti-mouse (#1706516, Bio-Rad, Hercules, CA, USA) HRP-conjugated secondary antibodies (1:5000) were incubated for 1 h at 4 °C. Membranes were washed as above in TBST for a minimum of 30 min, with a final wash in TBS. Membranes were incubated in Clarity ECL substrate (Bio-Rad, Hercules, CA, USA) and imaged using the Gel Doc imaging system (Bio-Rad, Hercules, CA, USA). Images were viewed using ImageJ.

### 4.7. Intracellular Viral Genome Quantitation

Intracellular viral genome quantitation was performed as previously described [36], and cells were infected as per ‘Cells and viruses”. At 12 and 120 HPI, cells were washed with PBS, lysed in TRIzol Reagent (Invitrogen, Waltham, MA, USA) on ice for 15 min, and the total DNA was isolated according to the manufacturer’s instructions. Two-step qPCR was performed using QuantiNova SYBR Green PCR master mix (Qiagen, Hilden, Germany) in a Rotor-Gene-Q real-time PCR cycler (Qiagen, Hilden, Germany). Initial denaturation was performed at 95 °C for 2 min, then 40 cycles of 30 s denaturation at 95 °C and 60 s anneal/extend at 60 °C. UL83 and ATG5 specific primers (Supplementary File 2) were used for HCMV genome quantification and as the internal control, respectively. Relative viral copy number was calculated using the ΔΔCt method, normalized to the 12 HPI control for both WT and ΔUL49 infections.

### 4.8. Immunofluorescence Confocal Microscopy

Cell monolayers were grown and infected on 12 mm glass coverslips (no. 1.5, Menzel, Waltham, MA, USA) in 24-well plates as per “Cells and Viruses.” Immunofluorescence confocal microscopy was performed as previously described [36]. Briefly, cells were fixed (4% PFA, PBS pH 7.4) and permeabilized (0.1% (*v*/*v*) Triton X-100 in PBS) for 15 min each, washed three times with 0.2% (*v*/*v*) Tween in PBS and blocked at RT for 1 h in blocking buffer (2% BSA, 0.2% Tween 20, 2.5% HSA in PBS). Primary mouse anti-IE1 (Clone 1B12 [82]) and anti-UL99 (Clone 10B4 [34]) were diluted 1:100. Additionally, mouse anti-GM130 (610822, BD Biosciences, Franklin Lakes, NJ, USA), rabbit anti-GM130 (ab52649, Abcam, Cambridge, UK), rabbit anti-HA epitope (3724T, Cell Signaling Technology, Danvers, MA, USA), mouse anti-lamin A/C (4777T, Cell Signaling Technology, Danvers, MA, USA), and rabbit anti-lamin B1 (ab16048, Abcam, Cambridge, UK) were diluted 1:500 in blocking buffer and incubated for 1 h at RT. Cells were washed three times in PBST and then incubated with Alexafluor 633 (A-21070, Invitrogen, Waltham, MA, USA) conjugated goat anti-rabbit IgG or Alexafluor 568 (A-11004, Invitrogen, Waltham, MA, USA) conjugated goat anti-mouse IgG diluted 1:1000 and Hoechst (33342, Thermo Fisher, Waltham, MA, USA) diluted 1:2000 for 30 min at RT. Coverslips were washed for 30 min in PBST, blotted dry on a Kimwipe, and mounted with Mowiol mounting media on glass slides (Menzel, Waltham, MA, USA). Images were acquired on a Leica (Wetzlar, Germany) inverted SP8 microscope using 63 × oil immersion objective and sequential scan settings of individual channels (Fluor 633, Fluor 568, eGFP, and Hoechst) with a line average of 4 and frame average of 1. Images were viewed in LAS X core offline version (Leica, Wetzlar, Germany).

### 4.9. RT-qPCR

MRC5 cultures were grown and infected at MOI = 3 as per “Cells and Viruses.” At 72 HPI, cells were lysed in TRIzol, and total RNA was extracted using the Direct-Zol MiniPrep kit (R2050, Zymo, Irvine, CA, USA), according to the manufacturer’s instructions. cDNA libraries were constructed using the SensiFAST cDNA synthesis kit (BIO-65054, Meridian Bioscience, Cincinnati, OH, USA) according to the manufacturer’s instructions. Two-step qPCR was performed using a Rotor-Gene-Q real-time PCR cycler (Qiagen, Hilden, Germany) and QuantiNova SYBR Green PCR master mix (Qiagen, Hilden, Germany). Initial heat denaturation was performed at 95 °C for 5 min, followed by 30 s denaturation at 95 °C. This was combined with anneal/extend for 60 s at 60 °C for 40 cycles. GAPDH was used as the internal control, and relative transcript abundance was quantified using the ΔΔCt method. All primer sequences are provided in Supplementary File S2.

### 4.10. High Pressure Freezing and Transmission Electron Microscopy

MRC5 monolayers were grown on carbon-coated 3mm sapphire disks (Engineering Office M. Wohlwend GmbH, Sennwald, Switzerland) and infected as per ‘Cells and Viruses.’ Cells were fixed with 4% paraformaldehyde in 1 × phosphate-buffered saline (PBS) for 15 min and rinsed in 1 × PBS. Sapphire disks were sandwiched between aluminium specimen carriers (Engineering Office M. Wohlwend GmbH, Sennwald, Switzerland) with hexadecane used as a filler and subjected to high-pressure freezing (HPF Compact 3, Engineering Office M. Wohlwend GmbH, Sennwald, Switzerland). Samples were freeze-substituted in 0.2% osmium tetroxide, 0.1% uranyl acetate, and 5% H_2_O in acetone using a Leica AFS2. After 24 h at −90 °C, the temperature was increased to 0 °C over 17 h. Samples were then removed from the AFS and left to reach room temperature for 20 min. Samples were rinsed three times in 100% acetone for 10 min prior to infiltration with increasing concentrations of Epon resin (25%, 50%, 75%, 100%, 100%, 100%), aided by a microwave regime (3 min for each step under vacuum at 250W, Pelco Biowave, Fresno, CA, USA). Sapphire discs were embedded in flat bottom capsules and polymerised for 48 h at 60 °C. After removal of the sapphire disk, cell monolayers were sectioned on a Leica UC7 ultramicrotome with a diamond knife (Diatome, Hatfield, PA, USA). 70 nm sections were collected on 100 mesh hexagonal copper grids and imaged by transmission electron microscopy at 80 kv (Jeol JEM 1400-Plus, Tokyo, Japan). A combination of single snapshots and image montages were collected of infected cells. Montages were automatically stitched by Jeol acquisition software (TEM Centre, Tokyo, Japan). Images were viewed and analysed using ImageJ.

### 4.11. RNA-seq

Cells were infected as per “Cells and virus” with MOI = 3. At 72 HPI, samples were lysed in TRIzol, and total RNA was extracted using the Direct-Zol MiniPrep kit (R2050, Zymo, Irvine, CA, USA), according to the manufacturer’s instructions. Sequencing libraries were prepared using an MGIEasy stranded mRNA kit and sequenced on an MGITech MGISEQ2000RS sequencer to generate 100 b paired-end readings.

Read data was aligned using the RNAsik pipeline (version 1.5.5 = 1) [85] to a custom reference genome containing human and HCMV sequences. The combined reference genome was generated by concatenating the fasta files from the X17403.1 reference for HCMV (downloaded from NCBI) and the GRCh38 Ensembl reference (release 104). The annotation GFF3 for X17403.1 was downloaded and modified to be compatible with the GRCh38 Ensembl GTF file before the two were concatenated to make a combined annotation GTF file. The RNAsik pipeline was run with the following parameters: ‘-align star -counts -paired -all’ and used the combined fasta file and gtf file as input for the ‘-fastaRef’ and ‘-gtfFile’ parameters. The RNAsik pipeline used STAR [86] to align the raw fastq files to the reference genome and featureCounts [87] to count high-quality, aligned reads to annotated genes. The resulting count matrix was then analysed for differential gene expression.

Data quality control, filtering, and differential expression (DE) analysis was performed using the DEBrowser Shiny user interface (v1.21.1) [88] in R Studio (v4.1.1). Low count filtering parameters were set to CPM < 3 in all eight samples and normalised using Trimmed Mean of M-values (TMM). Batch effect correction was set to “none.” DE analysis was performed between the 5 WT replicates and three replicates of ΔUL54 mutant samples with DE method: “Limma,” Normalization: “TMM,” Fit Type: “Is,” and Norm. Bet. Arrays: “none” set. The mean log_2_ fold changes for each gene were exported to Excel and used directly for further analysis and can be found in Supplementary File S1.

### 4.12. Bioinformatic Analysis

The relative abundance of each viral protein detected in the virion, and infected cell lysate [36] was log_2_ transformed. An enrichment score was calculated by dividing the virion abundance by cellular lysate abundance. For proteins detected in the cell lysate but not the virion, a score of -5 was imputed as this was the lowest real value calculated from the datasets. Kinetic expression class [19] and classification [37] were manually matched from the respective publications and Supplementary Files. For heatmaps, the Gower distance was calculated using the “daisy” function (cluster, Version 2.1.2) in R Studio (Version 1.4.1717) running R (Version 4.1.2). Heatmaps were constructed using ComplexHeatmap (Version 2.10.0) [89]. Complete Linkage was used as the clustering method, and the Gower distance data frame generated above was used for row clustering distance. The t-SNE analysis was performed in R using Rtsne (Version 0.15) with the Gower distance matrix as input. The t-SNE data output was overlaid with cluster information from the heatmap and plotted using ggplot2 (Version 3.3.5). The sub clustering (Figure 1B,C) was performed as above, with clusters I and II used as input for the Gower distance calculation.

For UL34 interaction analysis, UL34 and control raw spectral files were downloaded (ProteomeXchange, ID:PXD014845) [56] and analysed using MaxQuant (Version 1.6.0.13 [90]). Peptide spectra were searched using the Andromeda search engine integrated with MaxQuant [91] using a combined Uniprot reference comprising Human (Taxon ID:9606, 20,432 entries) and AD169 HCMV (Taxon ID: 10360, 193 entries) proteins. MaxQuant default search parameters were set with minimal changes. Methionine oxidation and N-terminal acetylation were set as ‘variable modifications,’ cysteine carbamidomethylation was set as a ‘fixed modification,’ and trypsin/P was selected as the ‘digestion enzyme.’ Additionally, 7 to 25 residues were set as “peptide length,” with a maximum number of two missed cleavages allowed. ‘Label-free quantification (LFQ)’ and ‘match between runs’ were enabled, and the protein false discovery rate was set to 1%. LFQ intensity was divided by the number of peptides detected for each protein before fold-change analysis. All data transformations were performed using Perseus [92]. LFQ values were log_2_ transformed, and missing values were imputed from the whole matrix distribution with a shrink factor of 0.3 and −1.8 standard deviation shift. For PCA, analysis was performed using ClustVis [93]. The Volcano plot was created in Perseus using the two-sided Student’s *t*-test, with a permutation-based false discovery rate of 0.05 and an S_0_ of 2 set. Cellular component gene enrichment analysis was performed using the GOrilla web tool (http://cbl-gorilla.cs.technion.ac.il/, accessed on 3 March 2022) [94] with significantly enriched UL34 interacting proteins as the target gene list. Term enrichment fold-change and *p*-values were plotted using ggplot2 (Version 3.3.5) in RStudio (Version 1.4.1717) running R (Version 4.1.2).

## Data Availability

The complete ΔUL54 RNA-seq dataset is available in the Gene Expression Omnibus (GEO) with identifier #GSE201208.

## Supplementary Materials

The following supporting information can be downloaded at: https://www.mdpi.com/article/10.3390/ijms23105773/s1.
