# Supplementary material for: UL34 Deletion Restricts Human Cytomegalovirus Capsid Formation and Maturation
Source: Int J Mol Sci. 2022 May 21;23(10):5773. doi: 10.3390/ijms23105773 (PMC9143689; doi:10.3390/ijms23105773)
Supplement: Supplementary file 1 [file ijms-23-05773-s001.zip › ijms-1699588-supplementary.pdf]

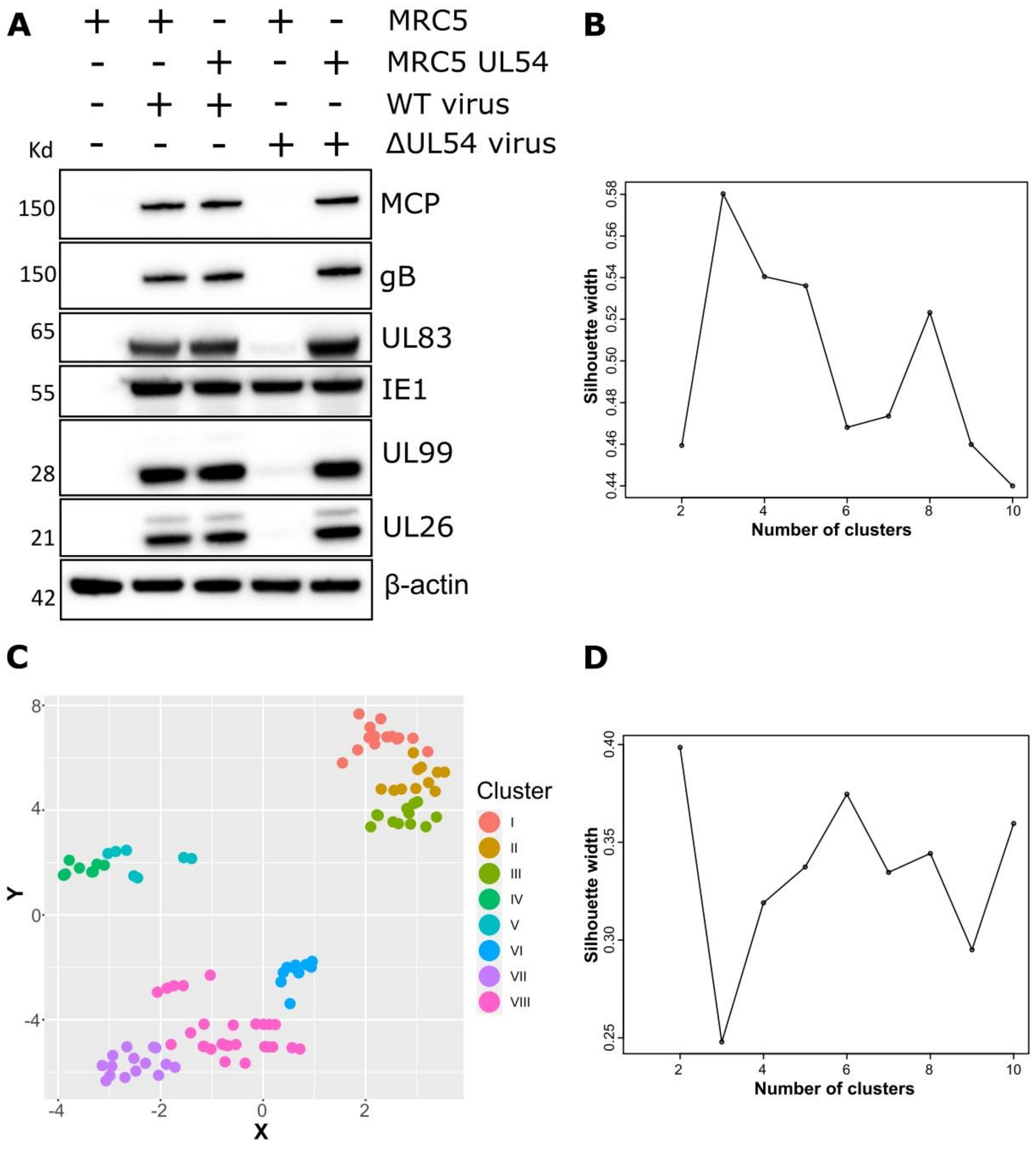

Figure S1. Assessment of ΔUL54 virus stock and hierarchical clustering. A) Western blot analysis of MRC5 and UL54 complementing cell line lysates infected with WT or ΔUL54 HCMV AD169-GFP viruses. 5 DPI, MOI = 3. Membranes were probed with primary antibodies against HCMV viral proteins or β-actin loading control. **B)** Line chart showing the silhouette widths for cluster number optimisation for Fig. 1A. **C)** t-SNE plot showing all HCMV genes analysed using Gower distance. Clusters I-VIII from Fig. 1A. **D)** Line chart showing the silhouette widths for cluster number optimisation for Fig. 1B.

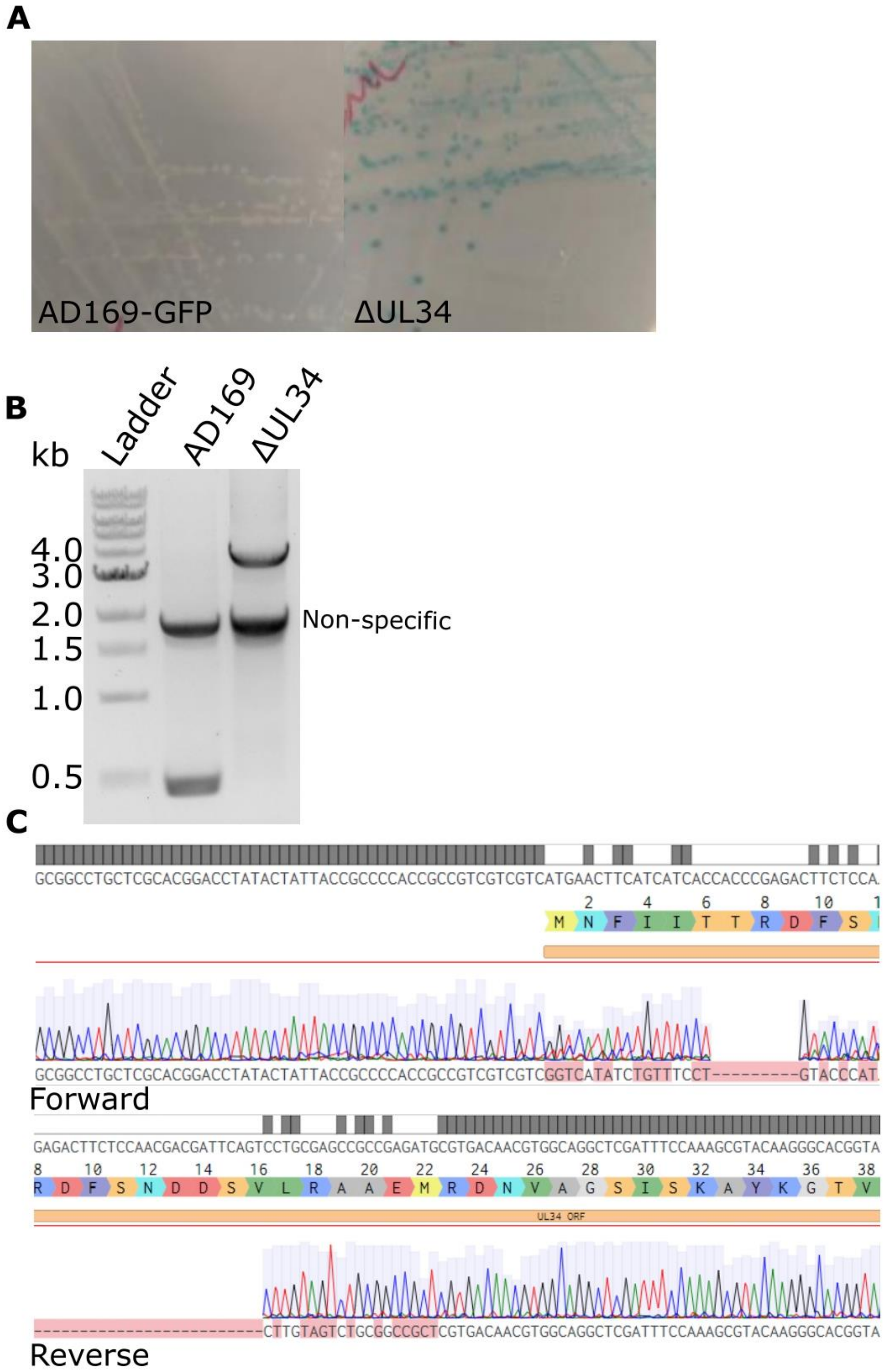

Figure S2. Validation of transposon insertion in ΔUL34 BAC. A) Blue-white validation of transposon insertion in ΔUL34 BAC. The transposon containing β-galactosidase metabolises X-gal to a blue product. **B)** Agarose gel showing PCR products for WT and ΔUL34 BAC templates. The F primer binds in the UL34 promoter region, and the R primer binds from nucleotide 574 of UL34. The 3.5 kb band in ΔUL34 indicates transposon insertion. **C)** Alignment of forward and reverse Sanger sequencing reads for the ΔUL34 PCR

product from B). The transposon deletes aa 1 to 22 in the n-terminus removing both possible translation start sites.

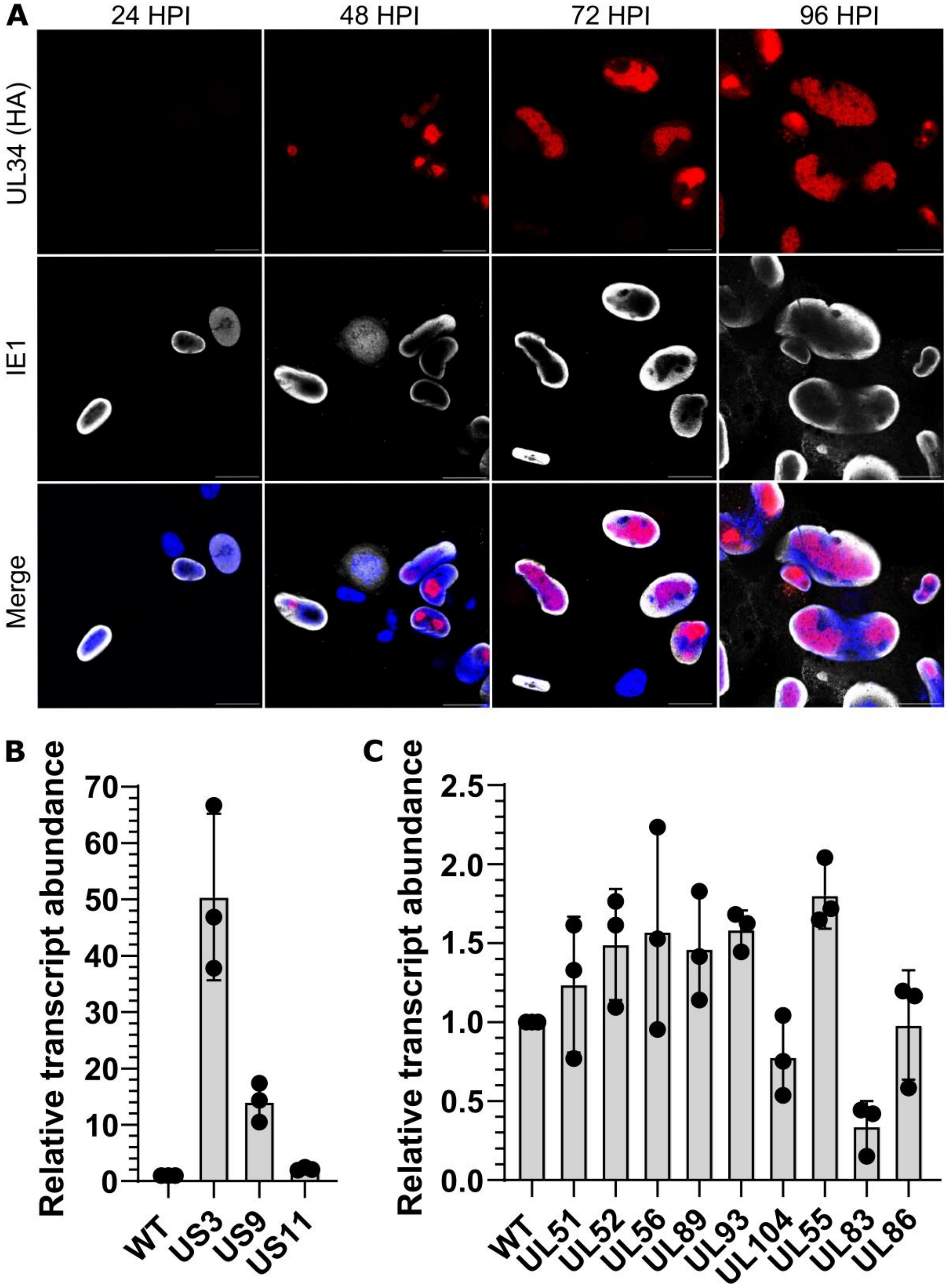

Figure S3. HA-UL34 localisation and RT-qPCR analysis in ΔUL34 infections. A) Immuno-fluorescence analysis of MRC5 cells infected with HA-UL34 AD169. Samples were fixed at 24, 48, 72 and 96 HPI, and stained with IE1 and HA (UL34) antibodies. MOI = 0.1,

scale bars = 20 µm. **B-C)** Relative abundance of select HCMV transcripts in HCMV AD169-GFP ΔUL34 infected cells compared to WT. MOI = 3, 72 HPI, n = 3, bars = SD.

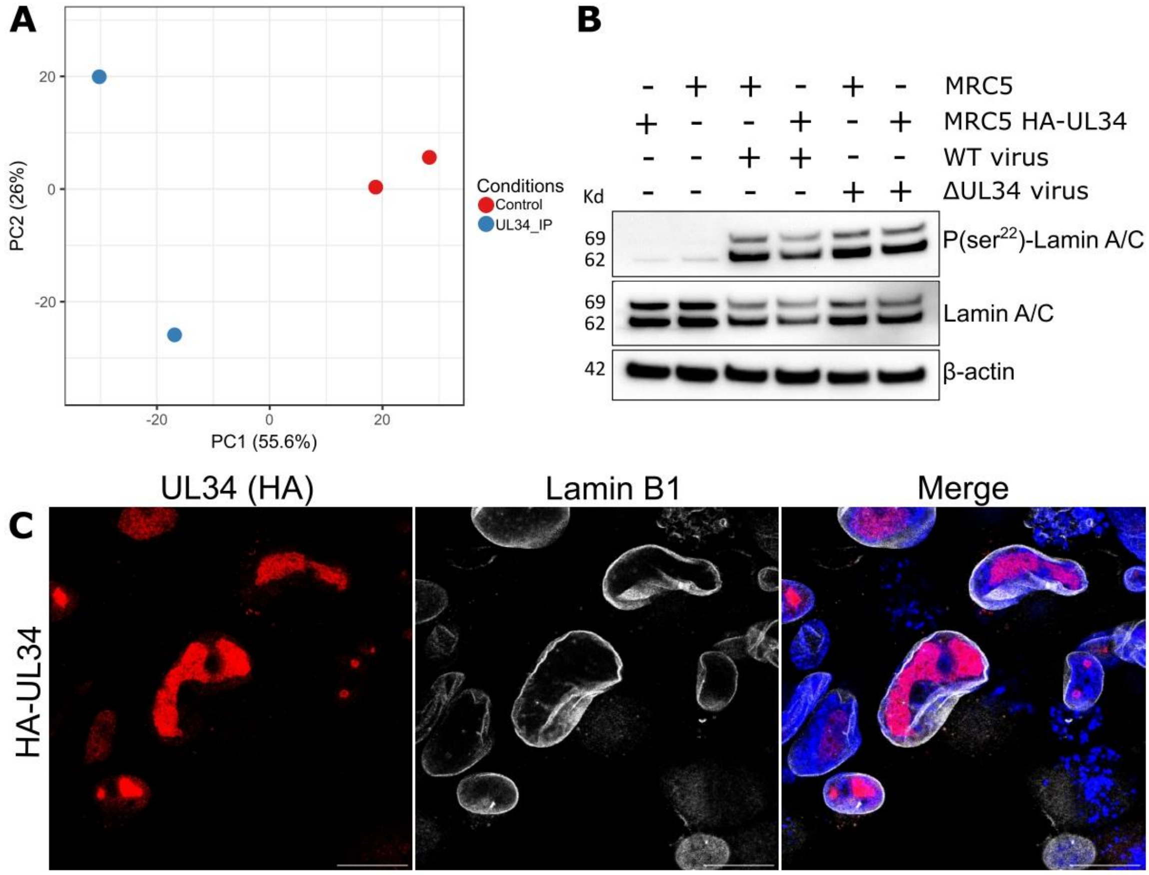

Figure S4. Characterisation of the host cell nuclear lamina in ΔUL34 infections. A) Principal component analysis plot of UL34 IP samples and control. Red: "GAW" control, blue: UL34_IP. **B)** Western blot analysis of MRC5 and UL34 complementing cell lysates infected with WT or ΔUL34 HCMV AD169-GFP viruses. 5 DPI, MOI = 3. Membranes were probed with primary antibodies against phospho-(ser^{22})-lamin A/C, lamin A/C or β-actin loading control. **C)** Immuno-fluorescence analysis of MRC5 cells infected with HA-UL34 AD169 or WT AD169-GFP. Samples were fixed and stained with lamin B1 and HA (UL34) antibodies. 4 DPI, MOI = 0.1, scale bars = 20 µm.
